# A novel role of NLRP3-generated IL-1β in the acute-chronic transition of peripheral lipopolysaccharide-elicited neuroinflammation: implications for sepsis-associated neurodegeneration

**DOI:** 10.1186/s12974-020-1728-5

**Published:** 2020-02-18

**Authors:** Zhan Zhao, Yubao Wang, Ran Zhou, Yi Li, Yun Gao, Dezhen Tu, Belinda Wilson, Sheng Song, Jing Feng, Jau-Shyong Hong, Jerrel L. Yakel

**Affiliations:** 1 Respiratory Department, Tianjin Medical University General Hospital, Tianjin, 300052 China; 2 Neurobiology Laboratory, National Institute of Environmental Health Sciences, National Institutes of Health, Research Triangle Park, NC 27709 USA; 3 Institute of Infectious Diseases, The Second Hospital of Tianjin Medical University, Tianjin, 300211 China

**Keywords:** Sepsis, Neuroinflammation, Acute-chronic transition, Neurodegeneration, NLRP3 inflammasome, IL-1β, IL-1R1, Parkinson’s disease

## Abstract

**Background:**

Sepsis-associated acute brain inflammation, if unresolved, may cause chronic neuroinflammation and resultant neurodegenerative diseases. However, little is known how the transition from acute to chronic neuroinflammation, which is critical for the following progressive neurodegeneration, occurs in sepsis. The goal of this study was to investigate potential immune factors regulating the transition process using a widely used endotoxemia LPS mouse model. This model shows distinct acute and chronic phases of neuroinflammation and recapitulates many cardinal features of Parkinson’s disease, thus, providing a unique opportunity for studying phase transition of neuroinflammation.

**Methods:**

C57BL/6 J, NLRP3^−/−^, and IL-1R1^−/−^ mice were employed. Mild and severe endotoxemia were produced by LPS ip injection at 1 or 5 mg/kg. Neuroinflammation in vitro and in vivo was assessed with proinflammatory cytokine expression by qPCR or ELISA and microglial activation by immunohistochemical analysis. Neurodegeneration was measured by manual and stereological counts of nigral dopaminergic neurons and immunohistochemical analysis of protein nitrosylation and α-synuclein phosphorylation.

**Results:**

LPS-elicited initial increases in mouse brain mRNA levels of TNFα, IL-6, IL-1β, and MCP-1, and nigral microglial activation were not dose-related. By contrast, the delayed increase in brain mature IL-1β levels was dependent on LPS doses and protracted nigral microglial activation was only observed in high dose of LPS-treated mice. LPS-elicited increase in brain mature IL-1β but not IL-1α level was NLRP3-dependent. After high dose LPS treatment, deficiency of NLRP3 or IL-1R1 did not prevent the initiation of acute neuroinflammation but abolished chronic neuroinflammation. Genetic or pharmacological inhibition of the NLRP3-IL-1β axis repressed LPS-stimulated upregulation of chronic neuroinflammatory mediators including MHC-II, NOX2, and Mac1, and protected dopaminergic neurons. Ten months after LPS-elicited severe endotoxemia, nigral persisted microglial activation, elevated nitrosylated proteins and phosphorylated α-synuclein, and significant neuronal degeneration developed in wild-type mice but not in NLRP3^−/−^ or IL-1R1^−/−^ mice.

**Conclusions:**

This study uncovers a novel role of the NLRP3-IL-1β signaling pathway in gauging the severity of sepsis-associated inflammation and determining whether acute neuroinflammation will resolve or transition to low grade chronic neuroinflammation. These findings also provide novel targets for developing therapy for severe systemic infection-related neurodegeneration.

## Background

Epidemiological investigations revealed that infections, such as sepsis (severe systemic infections) or viral infections, increase the risk of long-term brain dysfunction [1] and chronic neurodegeneration, including Parkinson’s disease (PD), Alzheimer’s disease (AD), and multiple sclerosis [2–7]. Symptoms of neurodegeneration in patients usually occur months or years after the incidence of infection. Strong evidence indicates an essential role of neuroinflammation in the pathogenesis of progressive neurodegeneration [8–16].

To model the sepsis-associated neuropathology, we have previously created a mouse model by a single intraperitoneal (ip) injection of sublethal dose of lipopolysaccharide (LPS, 5 mg/kg) [17–19]. Unlike most of acute PD animal models, which often kill neurons too fast [20], this model shows distinct acute and chronic phases of neuroinflammation and recapitulates many cardinal features of Parkinson’s disease, thus providing a unique opportunity for studying phase transition of neuroinflammation [21–23].

Numerous reports indicate that brain microglia are the proximal factor for sepsis-associated acute neuroinflammation. Once activated by sepsis via humoral pathway, neural pathway, and blood–brain barrier (BBB) alterations, microglia release a variety of cytokines, chemokines, and free radicals such as nitric oxide (NO) and reactive oxygen species (ROS) [24]. We have demonstrated that tumor necrosis factor alpha (TNFα) is necessary for initiating acute neuroinflammation after endotoxemia [17]. Although much is known about how endotoxemia affects brain immune functions, a crucial question as to how the transition of acute to chronic neuroinflammation occurs remains unanswered. The purpose of this study was to identify possible immune factors and investigate their mechanisms underlying this immune transition, which could offer new insights and potential therapies for sepsis-associated neurodegeneration.

Interleukin-1β (IL-1β), one of the potent proinflammatory cytokines, plays crucial roles in amplifying innate and adaptive immunity through its functional receptor IL-1 receptor 1 (IL-1R1) [25]; however, this cytokine could exacerbate tissue injuries in pathological conditions [26–32]. IL-1β precursor protein requires proteolytic cleavage by activated caspase-1 through the inflammasome, a large intracellular multiprotein complex, to generate mature IL-1β, the releasable and bioactive form [28, 30]. Nod-like receptor protein 3 (NLRP3) inflammasome can be activated by diverse stimuli, such as ATP, crystals, and endoplasmic reticulum (ER) stress, serving as a gate to produce mature IL-1β and involving multiple immune disorders [32–43]. The important role of NLRP3 inflammasome in mediating neurodegeneration produced by several toxins, including intracranial injection of LPS [44, 45], has been described. However, how precisely NLRP3-IL-1β impacts endotoxemia-elicited acut-chronic neuroimmune phase transition, which dominates the following progressive neurodegeneration, is still unknown. Thus, this study will provide the first evidence unveiling a novel role NLRP3-IL-1β axis in mediating sepsis-associated brain immune dynamic changes.

In this study, we investigated roles of the NLRP3-IL-1β signaling pathway in the transition of acute to chronic neuroinflammation using the peripheral LPS-induced mouse model [17, 21, 23]. We uncovered several unique changes of microglial NLRP3-generated IL-1β in response to LPS treatment in vitro and in vivo, including a delayed increase in the production and release of mature IL-1β compared with most other proinflammatory cytokines, and differential regulated precursor processing pattern in response to different doses of LPS. Moreover, genetic or pharmacological inhibition of either NLRP3 or IL-1R1 did not prevent the initiation of acute neuroinflammation but abolished long-term chronic neuroinflammation and subsequent neurodegeneration. To the best of our knowledge, this study provides the first evidence showing a novel role of NLRP3-IL-1β in mediating the transition from acute to chronic neuroinflammation incited by sepsis.

## Methods

### Animals

Male C57BL/6 J, NLRP3^−/−^, and IL-1R1^−/−^ mice at the age of 10- to 12-week-old were obtained from The Jackson Laboratory (Bar Harbor, Maine). All housing and breeding procedures and experimental protocols were approved by IACUC (Institutional Animal Care and Use Committee) of NIH (National Institutes of Health).

### Reagents

LPS (*Escherichia* coil O111:B4) used for cell culture studies was purchased from Calbiochem (San Diego, CA; cat# 437627) and for animal studies was purchased from Sigma-Aldrich (St. Louis, Mo; cat# L3012). Anti-tyrosine hydroxylase (TH) and anti-Iba-1 were purchased form EMD Millipore (Burlington, MA) and Wako (Richmond, VA), respectively. The secondary antibodies were purchased from Vector Laboratories (Burlingame, CA). The rat anti-mouse CD-11b antibody was purchased from abD Serotec (Raleigh, NC, cat# MCA711G). Anti-pro-IL-1β, anti-alpha-synuclein (α-synuclein), and anti-3-Nitrotyrosine (3-NT) antibodies were purchased from Abcam (Cambridge, MA). Mouse interleukin-1 receptor antagonist (IL-1Ra), NLRP3 inhibitor MCC950, caspase-1 inhibitor Z-YVAD, IRE1α (inositol-requiring enzyme 1α) inhibitor 4μ8C, TNF-α, and IL-1β ELISA kits were purchased from R&D Systems (Minneapolis, MN). Tauroursodeoxycholic acid (TUDC) was from Selleckchem (Houston, TX). Mouse IL-1β pro-form ELISA kit was from eBioScience (San Diego, California). Recombinant mouse IL-1β was from BioLegend (San Diego, CA). Cell culture ingredients were obtained from Invitrogen (San Diego, CA). All other reagents came from Sigma Chemical Co. (St. Louis, MO).

### Animal treatment

Mice, housed in a 12 h light/dark cycle for 1 week, received a single intraperitoneal injection of LPS [1 mg/kg (3 × 10^6^ EU/kg) or 5 mg/kg (15 × 10^6^ EU/kg)] or vehicle (PBS solutions). At different time points after LPS injection, mice were euthanized by Fatal-Plus overdose followed by cardiac perfusion with PBS (for mRNA and protein analysis) or formaldehyde (for IHC) and brains were collected. Brains for IHC were further post-fixed with 4% paraformaldehyde at 4 °C for 48 h, and subsequently immersed in 30% sucrose until the brains sank to the bottom of the container. Coronal sections (35 μm) encompassing SN pars compacta (SNpc) and hippocampus (Hip) were cut at 35 μm and stored in PBS.

### Primary mouse mesencephalic neuron-glial cultures

Neuron-glial cultures were prepared from the ventral mesencephalic tissues as previously described [46]. Ventral mesencephalic tissues were dissected from embryonic day 14 ± 0.5 and then dissociated with a mild mechanical trituration in ice-cold MEM. Cells were seeded to poly-d-lysine-coated 24-well (6.5 × 10^5^/well) plates with 0.5 ml/well of maintenance medium and place it in a humidified 37 °C, 5% CO_2_ incubator. Three days later, neuron-glia cultures were replenished with 0.5 ml/well fresh medium and were used for treatment at 7 days after their initial seeding. The composition of major cell types at the time of treatment was estimated by visual counting of immunostained cells with antibodies against cell-type specific markers: 11% microglia, 48% astrocytes, and 41% neurons, where approximately 1% of neurons were tyrosine hydroxylase-immunoreactive (TH-ir).

### Primary mouse mixed glial cultures

Primary mixed glial cultures were prepared by a previously described method [47]. Whole brains of postnatal day 1 neonates of C57BL/6 J mice, after stripping blood vessels and meninges, were dissociated by trituration in DMEM/F12 media. Cells were seeded to poly-d-lysine-coated 24-well (5.5 × 10^5^/well) plates with 0.5 ml/well of DMEM/F12 mixed glial culture media and maintained in a humidified 37 °C, 5% CO_2_ incubator. The medium was changed every 3 days with 1 ml/well of DMEM/F12 mixed glial culture media. Cultures were ready for treatment at 14 days after initial seeding. Based on the estimation by immunostained cells with specific microglia marker (Iba-1) and astrocyte marker (GFAP), mixed glia cultures contain about 20% microglia and 80% astrocytes.

### Immunohistochemistry and double-labeling immunofluorescence of brain slices

Free-floating 35 μm coronal brain slices encompassing SNpc and Hip regions were subjected to immunostaining as described previously [12]. After washing (two times) with PBS, the brain slices were treated with 1% hydrogen peroxide for 10 min. The slices were again washed (three times) with PBS and then incubated for 20 min with blocking solution (PBS containing 1% bovine serum albumin, 0.4% Triton X-100, and 4% appropriate serum to block the non-specific binding). The slices were incubated overnight at 4 °C with rabbit polyclonal antibody against tyrosine hydroxylase (TH) diluted (1:5000, Dopaminergic neuron marker), Iba-1 (1:5000, microglia marker), Phospho alpha-synuclein (S129) (1:5000), or rat monoclonal antibody against mouse CD11b (1:15000, microglia marker), in antibody diluents (DAKO), and then the slices were washed (three times) for 10 min each time in PBS. The slices were next incubated for 1 h with PBS containing 0.3% Triton X-100 and the appropriate biotinylated secondary antibody (goat anti-rabbit antibody, 1:227; horse anti-rat, 1:227). After washing (three times) with PBS, the slices were incubated for 1 h with the Vectastain ABC reagents (Vector Laboratory, Burlingame, CA) diluted in PBS containing 0.3% Triton X-100. To visualize the signal, the slices were incubated with 3,3′-diaminobenzidine and urea-hydrogen peroxide tablets dissolved in water. For morphological analysis, digital images were acquired under microscope (Nikon) connected to a Leica Aperio AT2 scanner.

For immunofluorescence, brain slices were incubated for 20 min in blocking solution to block non-specific binding. Slices were immunostained overnight at 4 °C with rabbit polyclonal antibodies against TH (1:5000) and co-labeled with a mouse monoclonal antibody against 3-nitrotyrosine (3-NT) (1: 1000). Antibodies were detected and visualized using Alexa Fluor 594 goat anti-rabbit IgG (1:750), or Alexa Fluor 488 goat anti-mouse IgG (1:750) secondary antibodies. The images were acquired using an inverted confocal microscope (Zeiss LSM 780). The intensities of Iba-1, CD-11b, Ser-129 phosphorylated α-synuclein, and immunofluorescence were analyzed using ImageJ software.

### Immunocytochemistry of cell cultures

Cultures were fixed with 3.7% formaldehyde in PBS for 20 min. After washing (two times) with PBS, the cultures were treated with 1% hydrogen peroxide for 10 min. The cultures were again washed (three times) with PBS and then incubated for 20 min with blocking solution. The fixed cultures were immunostained overnight at 4 °C with rabbit polyclonal antibodies against either TH (1:5000) or rat monoclonal antibody against mouse CD-11b (1:15000) in antibody diluents. Secondary antibody (1:227; Vector Laboratory) was added, amplified with Vectastain ABC reagents (Vector Laboratory), and visualized with 3,3′-diaminobenzidine (DAB). For morphological analysis and cell counting, images were acquired under microscope (Nikon) connected to a Leica Aperio AT2 scanner. The intensity of CD-11b was analyzed using ImageJ software.

### Real-time RT-PCR analysis

Total RNA was extracted from the mouse brain or cell cultures by using a RNeasy Mini Kit from QIAGEN (Valencia, CA) to detect the level of TNF-α, IL-1β, interleukin-6 (IL-6), monocyte chemoattractant protein-1 (MCP-1), major histocompatibility complex II (MHC-II), and NOX2 (gp91) according to the previous description [47]. Total RNA was reversely transcribed with MuLV reverse transcriptase and oligo-dT primers. Then SYBR green PCR master mix was used for real-time PCR analysis. The primer sequences were as follow: GAPDH F (5′ TTC AAC GGC ACA GTC AAG GC 3′), GAPDH R (5′ GAC TCC ACG ACA TAC TCA GCA CC 3′), TNF-α F (5′ GAC CCT CAC ACT CAG ATC ATC TTC T 3′), TNF-α R (5′ CCT CCA CTT GGT GGT TTG CT 3′), IL-1β F (5′ CTG GTG TGT GAC GTT CCC ATT A 3′), IL-1β R (5′ CCG ACA GCA CGA GGC TTT 3′), MHC-II F (5′ CCG TCA CAG GAG TCA GAA AGG 3′), MHC-II R (5′ CGG AGC AGA GAC ATT CAG GTC 3′), NOX2 F (5′ GAT TCA AGA TGG AGG TGG GAC 3′), NOX2 R (5′ GGT CAG TGT GAA TGG GTG CC 3′), MCP-1 F (5′ TTT GAA TGT GAA GTT GAC CCG 3′), and MCP-1 R (5′ GAA GTG CTT GAG GTG GTT GTG 3′). Real-time PCR amplification was performed using SYBR Green PCR Master Mix and QuantStudio 6 Flex Real-Time PCR System (Applied Biosystems, Foster City, CA, USA) according to manufacturer’s protocols. Amplifications were done at 95 °C for 10 s, 55 °C for 30 s, and 72 °C for 30 s for 40 cycles. All samples were tested in duplicate and normalized with GAPDH using the 2^-ΔΔCt^ method. Fold changes for each treatment were normalized to the control.

### Pro-IL-1β Western blot analysis

Tissues were homogenized in lysis buffer containing protease inhibitor cocktail. Equal protein from each half brain was separated by Bis-Tris-polyacrylamide electrophoresis gel, and then transferred to polyvinylidene difluoride (PVDF) membranes. Membranes were blocked with 5% non-fat milk and probed overnight at 4 °C with anti-pro-IL-1β antibody. Membranes were followed by incubating with secondary anti-rabbit IgG for 1 h. ECL Plus reagents were used as a detection system. Relative band intensities were quantified by ImageJ.

### Stereological and manual counting assessments of neurodegeneration

Dopaminergic neurons of the SNpc were identified through TH immunohistochemistry. The number of TH-immunoreacted (TH-ir) neurons in SNpc between saline and LPS-injected groups was counted with both optical fractionator and manual counting ways. For stereological counts, a total of eight sections containing the SNpc were sampled at 105-μm intervals. We used an optical fractionator method on an Olympus BX50 stereological microscope to estimate TH-ir neurons within user-defined boundaries [21–23]. Systematic random sampling of sites with an unbiased counting frame (100 μm × 100 μm) was conducted within defined boundaries of the SN. The guard zone was set at 2 μm and the dissector height was 11 μm. The counts were performed with an Olympus BX50 microscope with a 60 × 1.4NA oil immersion objective. The coefficient of error values was less than 0.1. For manual count, three individuals performed counting in a double-blind manner [46].

### Statistical analysis

Data was presented as the mean ± SEM. Comparison of more than two groups was performed using one-way ANOVA followed by Bonferroni post hoc multiple comparison test. Comparisons of more than two parameters were performed by two-way ANOVA analysis followed by Bonferroni post hoc multiple comparison test. Data were analyzed using Prism (v7.00, GraphPad, San Diego, CA). *P* values less than or equal to 0.05 were considered statistically significant.

## Results

### Brain-delayed mature IL-1β levels, but not initial acute proinflammatory response, are peripheral LPS dose-dependent

**Fig. 1:**
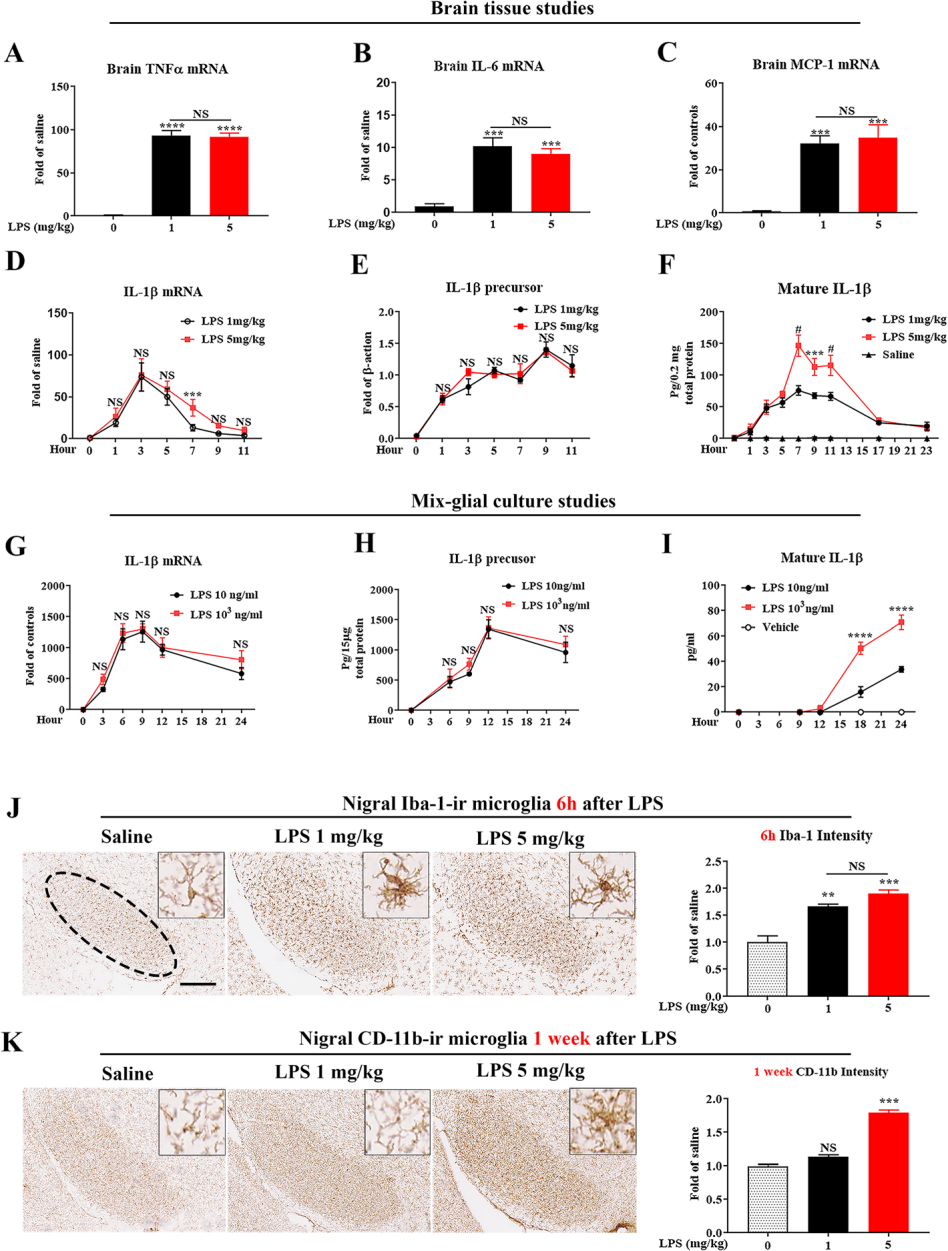
Peripheral LPS dose-dependently increases brain mature IL-1β production and causes sustained nigral microglial activation. In vivo cytokine measurements (**a**–**f**): At 1 h after injection of LPS (1 or 5 mg/kg, ip) or saline vehicle in C57BL/6 J mice, mRNA levels of TNFα (**a**), IL-6 (**b**), and MCP-1 (**c**) were detected by qPCR in brain tissues (*n* = 4/group). ***p < 0.001 and ****p < 0.001 compared with saline vehicle group. No significant difference (NS) between LPS 1 and 5 mg/kg groups. One-way ANOVA followed by Bonferroni post hoc multiple comparison test. Levels of IL-1β mRNA (**d**), and its precursor (**d**) and mature IL-1β (F) were measured in brain tissues at indicated time points by qPCR, Western blot quantification, and ELISA, respectively (*n* = 3–5/group for each timepoint). ***p < 0.001, #*p* < 0.0001, or NS compared with LPS 1 mg/kg group. Two-way ANOVA followed by Bonferroni post hoc multiple comparison test. In vitro IL-1β measurements (**g**–**i**): In C57BL/6 J mix-glia cultures after LPS (10 ng/ml or 10^3^ ng/ml), or vehicle medium treatment, mRNA (**g**), supernatant precursor (**h**), and mature form (**i**) of IL-1β were determined at indicated time points by qPCR or ELISA, respectively. Results were from three independent experiments. ****p < 0.001 compared with LPS 10 ng/ml group. Two-way ANOVA followed by Bonferroni post hoc multiple comparison test. In vivo microglial activation (**j**, **k**): At 6 h and 1 week after injection of LPS (1 or 5 mg/kg, ip) or saline vehicle in C57BL/6 J mice (*n* = 3/group for each timepoint), nigral microglial Iba-1 (**j**) or CD-11b (**k**) immunostaining was performed, respectively. Representative images were shown. Scale bar = 300 μm. The histograms showed the density of Iba-1 (**j**) or CD-11b (**k**) quantified by with ImageJ. **p < 0.01 and ***p < 0.001 compared with saline vehicle group, and NS between LPS 1 and 5 mg/kg groups. One-way ANOVA followed by Bonferroni post hoc multiple comparison test

Mice received a single intraperitoneal injection of either 1 or 5 mg/kg of LPS to produce either mild or severe endotoxemia. No difference in the increases in brain mRNA levels of TNFα, IL-6, MCP-1, and IL-1β were observed between LPS 1 and 5 mg/kg groups at 1 h after injection, indicating that brain initial proinflammatory responses are not related to LPS doses (Fig. 1a–d). Different from most proinflammatory cytokines, the production of mature IL-1β is inflammasome-dependent via limited proteolysis of its precursor protein. Therefore, we compared time-course expression of not only IL-1β mRNA and precursor but also IL-1β mature form, to determine whether endotoxemia severity affects brain mature IL-1β levels. We found that 1 and 5 mg/kg of LPS-elicited increases in IL-1β mRNA and precursor levels are similar (Fig. 1d, e and Additional file 1: Figure S1). By contrast, high dose of LPS produced much higher brain mature IL-1β than that of low dose of LPS during 7–11 h after LPS injection (Fig. 1f). After 24 h, brain mature IL-1β concentrations were too low to detect by the ELISA kit.

For more detailed mechanistic studies on the processing of IL-1β protein, we employed primary mouse mix-glial cultures, which mainly contain 20% microglia and 80% astroglia. There are two reasons for using mix-glial cultures, instead of enriched microglial cultures. First, reports indicate that microglia are the main source of IL-1β production in the brain after peripheral LPS injection [48–50] and in cell cultures upon LPS stimulation [39]. Second, the presence of astroglia helps the survival and stabilization of microglia in cultures [51]. Two concentrations of LPS (10 and 1000 ng/ml), which produce different degrees of inflammatory responses, were added to the cultures. And then IL-1β mRNA, precursor protein, and mature IL-1β were measured at different times afterwards. Similar to brain tissues, mix-glial cultures produced comparable increases in the expression of IL-1β mRNA and precursor levels between high and low concentrations of LPS treatment (Fig. 1g, h). By contrast, high concentrations of LPS caused a larger delayed increase in the supernatant IL-1β level than that of low concentrations of LPS (Fig. 1i).

### High dose, but not low dose, of peripheral LPS causes long-lasting nigral microglial activation

In vitro and in vivo findings prompted us to further determine whether the dose-related increase in mature IL-1β could be related to the transition of acute to chronic inflammation. To obtain evidence supporting this hypothesis, we investigated the persistence of microglia activation after 1 and 5 mg/kg LPS treatment. For monitoring microglial activation, immunostaining of Iba-1 (ionized calcium binding adaptor molecule 1) was performed at acute stage (6 h after LPS) and CD-11b (α-chain of Mac1 receptor) was used as a marker for the chronic stage (1 week after LPS). The reason for using two different markers at different stages is based on our previous reports indicating that microglial Iba-1 but not CD-11b expression was acutely enhanced after LPS injection. During the acute inflammation stage, the level of CD-11b at 6 h after LPS was too low for immunostaining. By contrast, the increase of CD-11b expression was much higher than that of Iba-1 at 1 week and later time points [21, 52]. In this study, Iba-1 staining quantification revealed that acute microglial activation in the substantia nigra was apparent at 6 h after LPS injection, but there were no differences between LPS 1 and 5 mg/kg groups (Fig. 1j). One week later, nigral microglial morphology in 1 mg/kg LPS group was similar to that of saline group, suggesting that acute neuroinflammation had resolved (Fig. 1k). In contrast, protracted neuroinflammation occurred in 5 mg/kg LPS group, which showed clear sustained activation of microglia in the SN region (Fig. 1k). Thus, brain-delayed mature IL-1β levels and prolonged neuroinflammation are both affected by LPS in a dose-dependent manner, which is consistent with the idea that IL-1β may serve as a critical factor in the transition from acute to chronic neuroinflammation.

### Deficiency of NLRP3 reduces LPS-elicited increase in IL-1β, but not IL-1α, levels

**Fig. 2:**
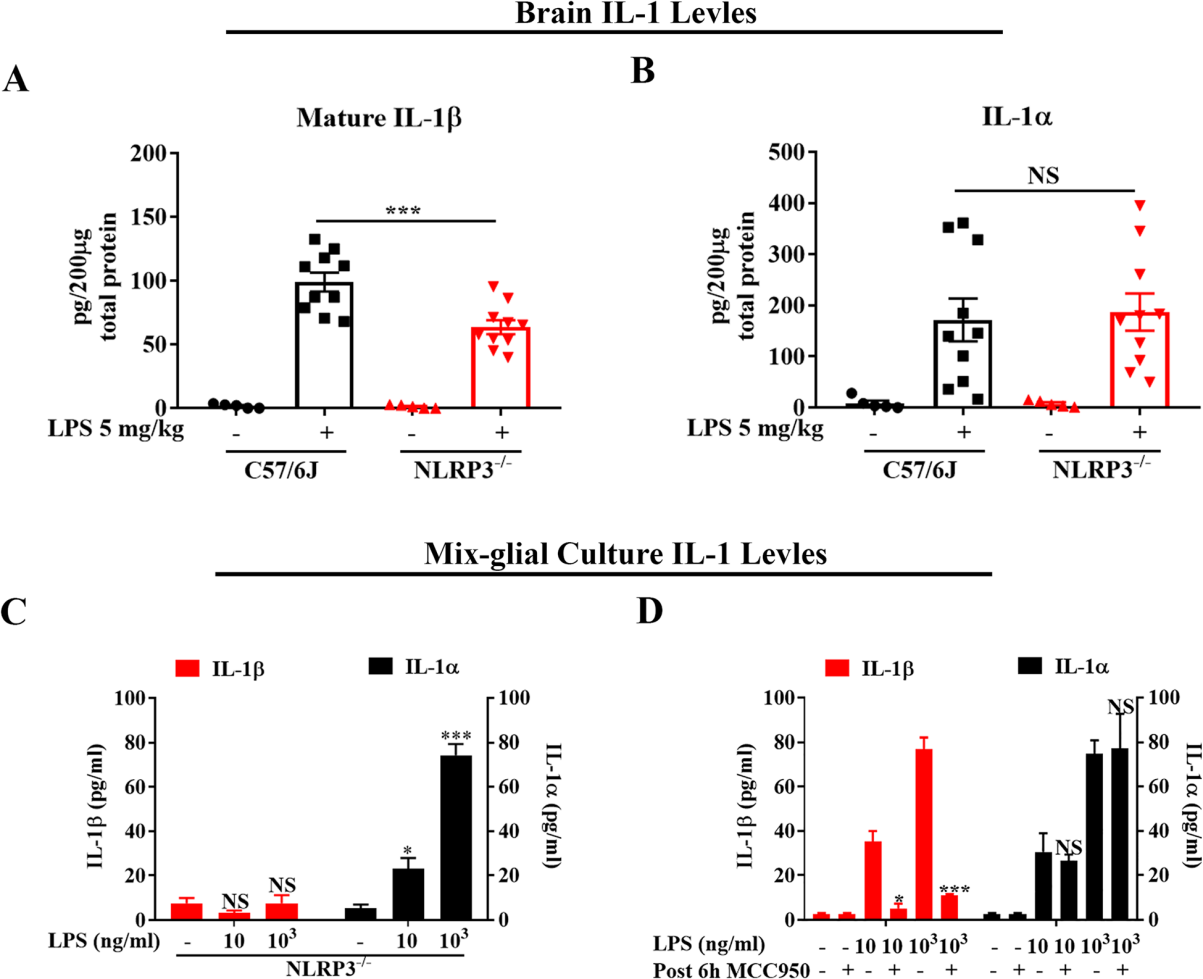
Lacking NLRP3 lessens endotoxemia-elicited increase in IL-1β, but not IL-1α, production. At 9 h after LPS 5 mg/kg or saline ip injection, levels of mature IL-1β (**a**) and IL-1α (**b**) were measured in 0.2 mg total protein from brain homogenization of WT and NLRP3^−/−^ mice by ELISA (*n* = 5–10/group). ***p < 0.001 for (**a**); NS for (**b**) between LPS-treated WT and NLRP3^−/−^ groups. Two-way ANOVA followed by Bonferroni post hoc multiple comparison test. **c** Levels of IL-1β and IL-1α in the supernatant of NLRP3^−/−^ mix-glial cultures at 24 h after vehicle or LPS treatment. Data were from three independent experiments. *p < 0.05, ***p < 0.001, and NS compared to respective vehicle medium group. One-way ANOVA followed by Bonferroni post hoc multiple comparison test. **d** Mix-glial cultures were treated with 10 ng/ml or 10^3^ ng/ml of LPS or vehicle medium. MCC950 (10 μM) were added to cultures 6 h later. Culture supernatant levels of IL-1β and IL-1α were assessed at 24 h after LPS treatment. Data were from three independent experiments. *p < 0.1, ***p < 0.001, and NS compared with the same dose of LPS treatment group without MCC950. Two-way ANOVA followed by Bonferroni post hoc multiple comparison test

Among inflammasome family members, NLRP3 inflammasome is abundantly expressed and plays a key role in IL-1β processing in the brain [53–55]. However, whether elevated brain IL-1β levels incited by endotoxemia are NLRP3-dependent remain unclear. Since brain mature IL-1β levels peaked around 7–11 h after LPS injections (Fig. 1f), we compared 9 h brain mature IL-1β between NLRP3-deficient (NLRP3^−/−^) and WT mice and found that NLRP3^−/−^ mice generated significantly less brain mature IL-1β than that of WT mice (Fig. 2a). In mix-glial cultures, either genetic knockout of NLRP3 or pharmacological inhibition of NLRP3 by MCC950 (an NLRP3 inhibitor) [56] significantly reduced LPS-stimulated mature IL-1β release (Fig. 2c, d). We also measured LPS-elicited increase in brain and culture supernatant levels of IL-1α, another IL-1 family member, which is the ligand of IL-1R1 too [29], to determine whether processing of this cytokine is NLRP3-dependent. LPS-elicited IL-1β levels were decreased both in brain tissues and the supernatant of mix-glial cultures in the absence of NLRP3. By contrast, brain IL-1α productions were independent of NLRP3 (Fig. 2b, c). Similarly, post-treatment of MCC950 reduced the production of IL-1β but not IL-1α (Fig. 2d).

### ER stress mediates LPS alone-induced NLRP3-dependent IL-1β production

We first determined whether LPS enhances the expression of NLRP3 and found that mRNA levels of NLRP3 were greatly increased and peaked at 3 h after LPS treatment in mixed glial cultures (Additional file 2: Figure S2a). Additional studies showed that YVAD (a caspase-1 inhibitor) significantly repressed LPS-induced increase in the release of mature IL-1β (Additional file 2: Figure S2b). These results indicate the involvement of the NLRP3-caspase-1 pathway in LPS alone-induced microglial mature IL-1β release. In fact, we have performed Western blot analysis to measure levels of NLRP3, caspase-1 (in pro and mature form), but not ASC (antibody used did not work), in both microglia-enriched cultures and in intact brains and dose-dependent increases in both NLRP3 and mature form of caspase-1. LPS-elicited increase in activated caspase-1 and mature IL-1β levels were significantly reduced in the presence of the NLRP3 inhibitor MCC950 in primary microglia-enriched cultures. These data were included in a separate paper submitted to another journal for publication (Gao et al., submitted for publication).

Most investigations have demonstrated that NLRP3 inflammasome-dependent processing of IL-1β precursor to mature form requires secondary stimulating agents, such as ATP, or pore-forming toxins, to activate the NLRP3-caspase-1 pathway [39]. Interestingly, LPS alone in our study can trigger microglial mature IL-1β release in the absence of known NLRP3 activators. We were wondering the mechanism underlying LPS alone-induced microglial IL-1β maturation. Furthermore, a recent report indicated that ER stress can activate NLRP3 inflammasome in macrophage stimulated by pathogen alone in the absence of NLRP3 inflammasome activators [43]. Additionally, increased levels of ER stress makers were observed in the brain after LPS ip injection and relief of ER stress decreased brain cytokine production including IL-1β [57]. Therefore, we investigated a potential role of ER stress in mediating LPS alone-induced NLRP3-dependent mature IL-1β production in microglia. Our data showed that LPS-induced mature IL-1β levels declined significantly by post-treatment with ER stress inhibitors either tauroursodeoxycholic acid (TUDC, a chemical chaperon mitigating ER stress) or 4μ8C (a specific IRE1α inhibitor), suggesting a role of ER stress in LPS alone-induced microglial activation of NLRP3 inflammasome (Additional file 2: Figure S2c).

### The NLRP3- IL-1R1 pathway dictates peripheral LPS-elicited transition from acute to chronic neuroinflammation

**Fig. 3:**
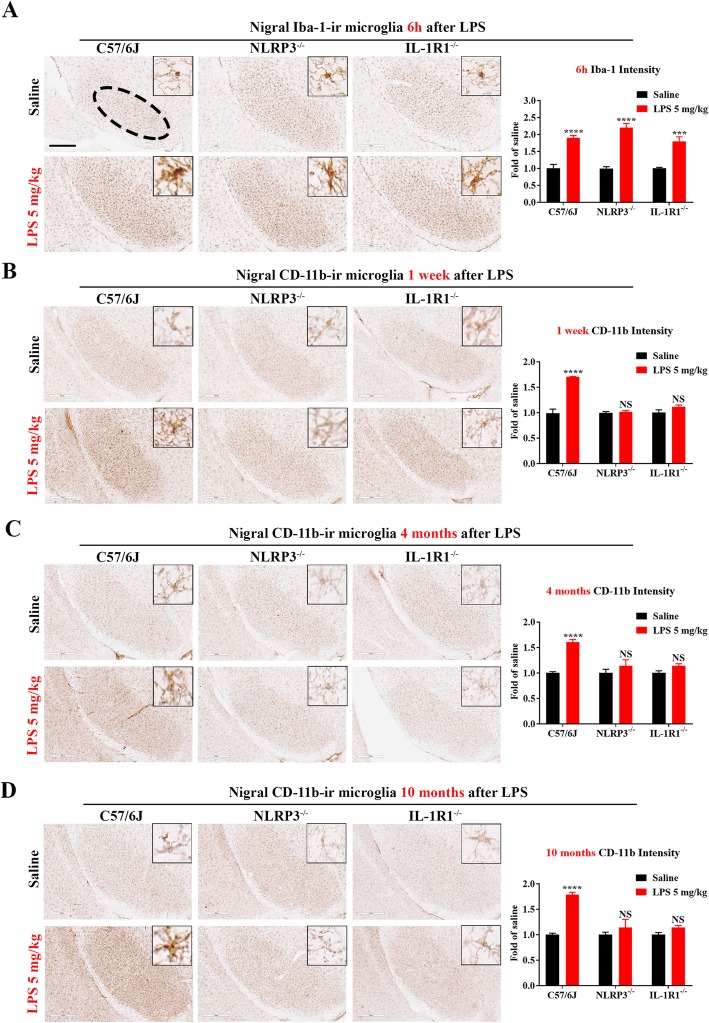
High dose LPS injection produces long-lasting microglial activation in WT mice, but not in NLRP3^−/−^ or IL-1R1^−/−^ mice. Following a single injection of LPS (5 mg/kg; ip) or saline vehicle, mice were perfused 6 h, 1 week, 4 and 10 months thereafter for nigral microglial Iba-1 or CD-11b immunostaining (*n* = 3/group for each time point). Representative images at each time point were shown (**a**–**d**). Scale bar = 300 μm. Results are expressed as folds of time-matched vehicle control. Histograms represent degree of microglial activation quantified by measuring the density of Iba-1 or CD-11b staining with ImageJ. ***p < 0.001, ****p < 0.0001, and NS compared to respective saline vehicle group. Two-way ANOVA followed by Bonferroni post hoc multiple comparison test

IL-1β acts on two receptors: IL-1R1, a receptor responsible for IL-1β signal transduction via MyD88-NF-κB and MAPK pathways, and IL-1R2, a decoy receptor that blocks the function of IL-1β [25]. To further elucidate the role of IL-1β in LPS-induced acute-chronic transition of neuroinflammation, we injected wild-type (WT), NLRP3^−/−^, and IL-1R1^−/−^ mice with LPS (5 mg/kg, ip). This dose of LPS produced prolonged neuroinflammation in WT mice (Fig. 1k). Increased levels of brain mRNA including TNFα, IL-6, and MCP-1 measured at 1 h after LPS injection were similar among these three strains of mice (Additional file 3: Figure S3a-c), except the IL-1β mRNA levels, which were slightly higher in NLRP3^−/−^ mice than those in WT mice, perhaps due to a compensatory effect (Additional file 3: Figure S3d). Furthermore, during the acute phase of LPS-elicited neuroinflammation (6 h after LPS), no difference in microglial activation as shown by enhanced Iba-1 immunoreactivity in the SN region was observed between these three strains of mice (Fig. 3a). Thus, sepsis still launches acute proinflammatory response in the brain without the participation of NLRP3 or IL-1R1. By contrast, NLRP3 and IL-1R1 are required for long-term maintenance of microglial activation after sepsis. During the chronic neuroinflammatory phase (1 week, 4 and 10 months after LPS), higher levels of CD-11b immunoreactivity and activated morphology of nigral microglia were still evident in WT mice, but not in both NLRP3- or IL-1R1-deficent mice (Fig. 3b–d). Taken together, our results from different doses of LPS (Fig. 1), plus data from mutant mice (Figs. 2 and 3), strongly indicate a critical role of the NLRP3-IL1β-IL-1R1 pathway in mediating the transition from acute to chronic neuroinflammation incited by severe endotoxemia.

### Genetic/pharmacological inhibition of NLRP3 or IL-1R1 represses LPS-elicited production of chronic inflammatory mediators in neuron-glial cultures

To determine potential inflammatory factors mediating NLRP3-IL-1β-elicited chronic neuroinflammation, we performed in vitro studies using mouse primary midbrain neuron-glial cultures containing approximately 40% neurons, 10% microglia, and 50% astroglia. To observe clearer neuronal damage and reactive microgliosis, we treated neuron-glial cultures with a higher concentration of LPS (20 ng/ml). In this series of studies, we assessed the consequence of lacking either NLRP3 or IL-1R1 on the induction of acute factors such as TNFα, and delayed factors including MHC-II, NOX2 (gp91), and Mac1. These delayed factors are necessary for sustained reactive microgliosis and long-lasting neuroinflammation [11, 21, 58–67].

**Fig. 4:**
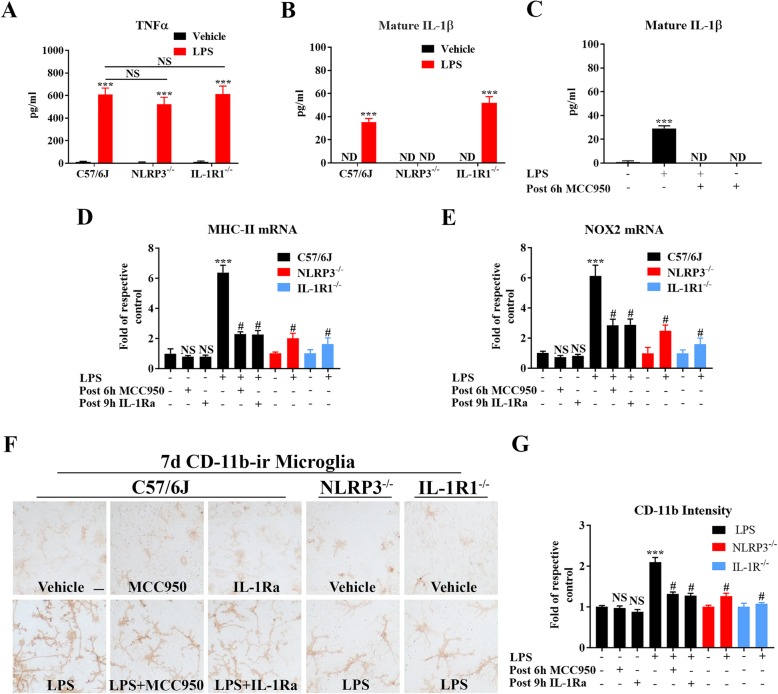
Genetic or pharmacological inhibition of NLRP3 or IL-1R1 represses LPS-elicited production of chronic inflammatory mediators in neuron-glial cultures. Neuron-glial cultures prepared from WT, NLRP3^−/−^, and IL-1R1^−/−^ mice were treated with LPS (20 ng/ml) or vehicle medium. Data were from three independent experiments. After LPS treatment, supernatant levels of 4 h TNFα (**a**), 24 h mature IL-1β (**b**), and 24 h mature IL-1β with post 6 h addition of MCC950 at 10 μM (**c**) were detected by ELISA. ***p < 0.001 compared with respective vehicle medium group and NS among LPS groups. Two-way ANOVA followed by Bonferroni post hoc multiple comparison test for (**a**, **b**). One-way ANOVA followed by Bonferroni post hoc multiple comparison test for (**c**). At 4 days after LPS, mRNA levels of MHC-II (**d**) and NOX2 (**e**) were measured by qPCR. MCC950 (10 μM) or IL-1Ra (0.5 μg/ml) was post-added to WT neuron-glial cultures at 6 h and 9 h. *ND* not detectable. At 7 days after LPS and post-treatment of MCC950 or IL-1Ra, representative images of microglial CD-11b (the α-chain of Mac1, indicating Mac1 expression here) immunostaining (**f**) and the intensity of CD-11b (**g**) measured by ImageJ were shown. Bar = 50 μm. **d**–**g** NS, #*p* < 0.001 compared with WT vehicle group, and ***p < 0.001 compared with WT LPS group. Two-way ANOVA followed by Bonferroni post hoc multiple comparison test

We first demonstrated that the lack of NLRP3 or IL-1R1 did not impede the initiation of LPS-elicited acute immune response. LPS stimulated a large increase of TNFα level measured at its peak release level (4 h after LPS) in cultures prepared from WT, NLRP3^−/−^, and IL-1R1^−/−^ mice; however, no difference in the amount of increase was observed among these three groups (Fig. 4a). There was a delayed increase in the release of IL-1β in WT and IL-1R1^−/−^, but not in NLRR3^−/−^ cultures (Fig. 4b). Furthermore, addition of a NLRP3 inhibitor MCC950 to the cultures diminished LPS-elicited IL-1β production (Fig. 4c).

Several late-expressed genes, including MHC-II, NOX2, and Mac1, which are involved in mediating chronic neuroinflammation were determined using NLRP3 or IL-1R1 inhibitors. To avoid interfering with LPS-elicited acute neuroinflammatory response, MCC950 was added 6 h and an IL-1 receptor antagonist (IL-1Ra) was added 9 h after LPS treatment. These two posttreatment time points reflect the peak of IL-1β processing and the peak of IL-1β release, respectively. The blockade of the NLRP3-IL-1β pathway by either preventing the production or inhibiting the action of IL-1β greatly reduced LPS-induced upregulated expression of MHC-II and NOX2 mRNA levels and immunoreactivity of CD-11b during the chronic phase of neuroinflammation (Fig. 4d–g). Together, results from in vitro studies are consistent with our in vivo data and further support the concept that the NLRP3-IL-1β signaling pathway is essential in mediating the transition of acute to chronic neuroinflammation.

### Genetic mutation or pharmacological inhibition of NLRP3 or IL-1R1 prevents LPS-elicited dopaminergic neurons death

Strong emerging evidence supports the notion that sustained neuroinflammation drives chronic neurodegeneration as mentioned in the introduction. Since the deficiency of NLRP3-IL-1β pathway prevented chronic, but not acute, neuroinflammation after sepsis (Figs. 3, 4, and Additional file 2: Figure S2), we next studied whether blockage of generation or action of IL-1β would avoid sepsis-associated neurodegeneration once acute neuroinflammation began.

**Fig. 5:**
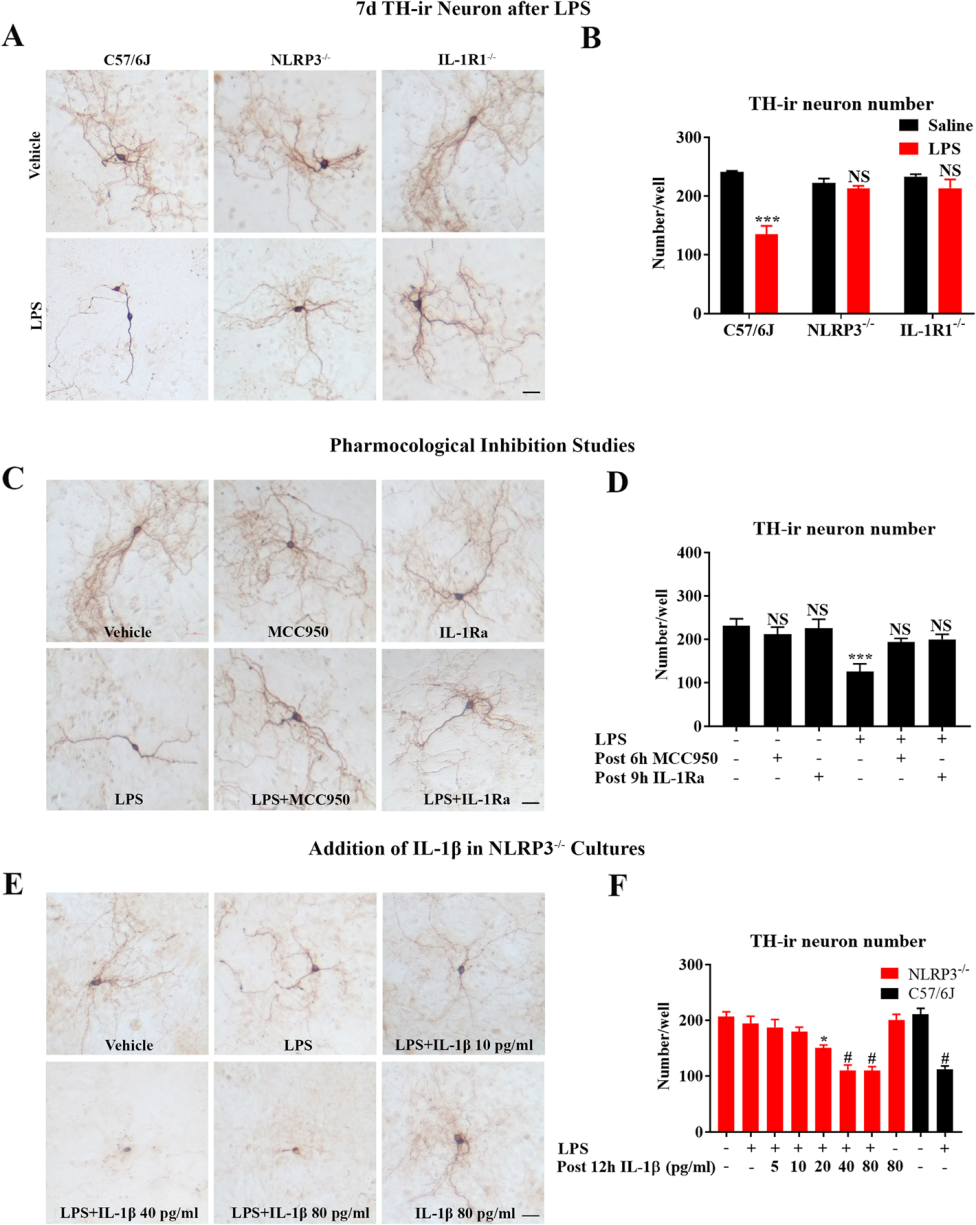
Genetic or pharmacological inhibition of NLRP3 or IL-1R1 does not affect TNFα release but protects dopaminergic neurons against LPS-induced toxicity in neuron-glia cultures. Neuron-glial cultures prepared from WT, NLRP3^−/−^, and IL-1R1^−/−^ mice were treated with LPS (20 ng/ml) or vehicle medium. Data were from three independent experiments. Scale bar = 50 μm. At 7 days after LPS, representative images of TH immunostaining (dopaminergic neurons) (**a**) and TH-ir neuron number (**b**) were shown. At 7 days after LPS injection and post-treatment of MCC950 (10 μM at 6 h) and IL-1Ra (0.5 μg/ml at 9 h), representative images of TH immunostaining (**c**) and TH-ir neuron number (**d**) were shown. **b**, **d** ****p < 0.0001 and NS compared with respective vehicle medium group. Two-way ANOVA followed by Bonferroni post hoc multiple comparison test for (**b**) and one-way ANOVA followed by Bonferroni post hoc multiple comparison test for (**d**). At 7 days after LPS and post 12 h addition of different amounts of recombinant mouse IL-1β to NLRP3^−/−^ neuron-glial cultures, representative images of TH immunostaining (**e**) and TH-ir neuron number (**f**). *p < 0.5 and #*p* < 0.0001 compared with respective vehicle group. One-way ANOVA followed by Bonferroni post hoc multiple comparison test

**Fig. 6:**
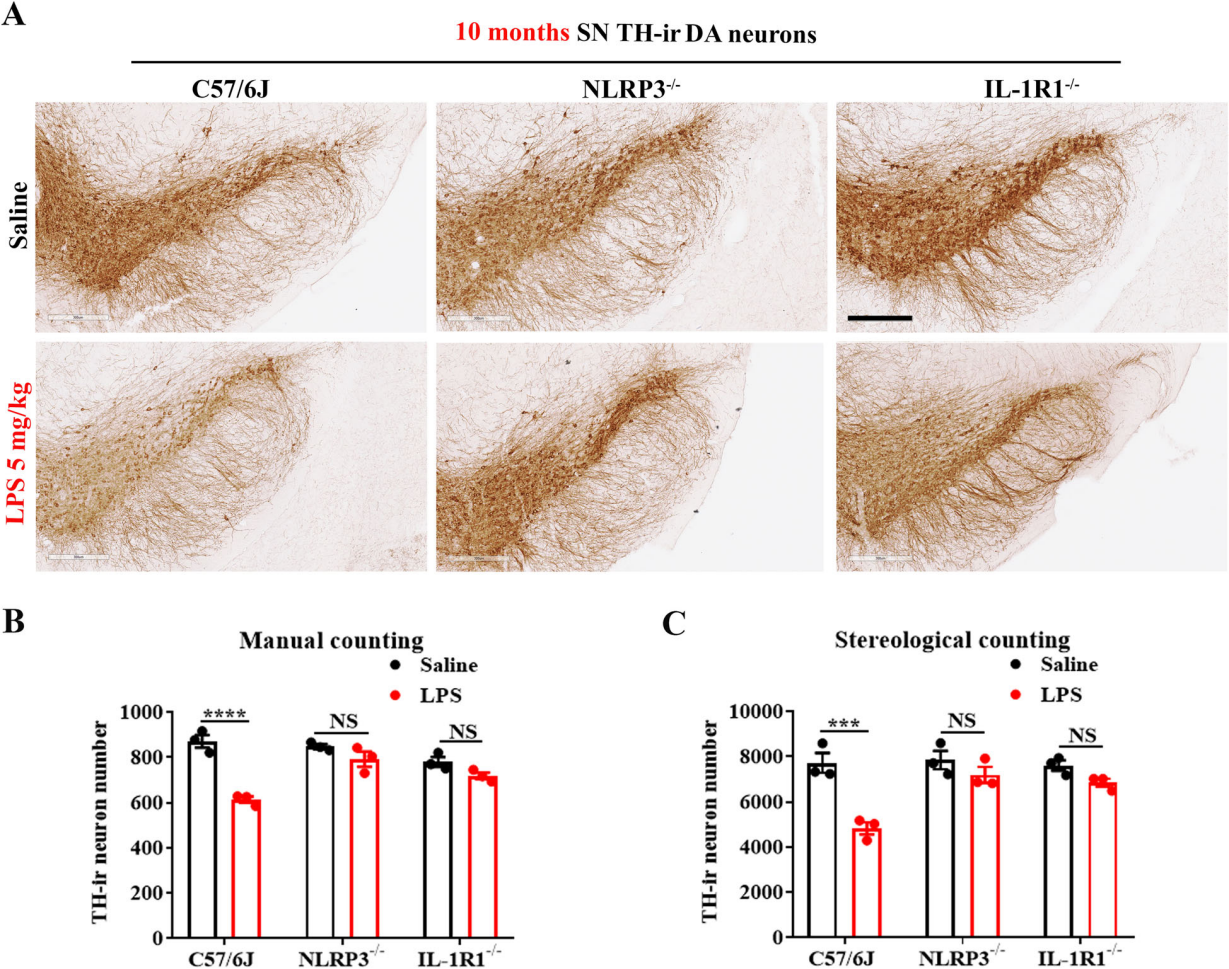
Endotoxemia-elicited neurodegeneration is ameliorated in NLRP3^−/−^ or IL-1R1^−/−^ mice. Representative images of TH immunostaining (**a**) in SN of WT, NLRP3^−/−^, and IL-1R^−/−^ mice at 10 months after LPS 5 mg/kg or saline i.p injection were shown (*n* = 3/group). Scale bar = 300 μm. Brain TH-ir neuron number was manually (**b**) and stereologically (**c**) counted, respectively, after LPS or vehicle treatment indicated in picture (**a**). ***p < 0.001 and NS compared with respective saline group. Two-way ANOVA followed by Bonferroni post hoc multiple comparison test

We first performed an in vitro study by using mouse primary midbrain neuron-glia cultures prepared from WT, NLRP3^−/−^, or IL-1R1^−/−^ mice as described above. A 40–50% tyrosine hydroxylase (TH)-immunoreactive (ir) neurons (dopaminergic neurons) loss was found at 7 days after LPS treatment in WT cultures. By contrast, LPS failed to produce significant loss of dopaminergic neurons in either NLRP3^−/−^ or IL-1R1^−/−^ cultures (Fig. 5a, b). Next, following LPS treatment, we post-treated cultures with MCC950 at 6 h or IL-1Ra at 9 h after LPS (Fig. 4d, e). The results showed that 7 days later, LPS-elicited loss of TH-ir neurons was prevented by these two inhibitors (Fig. 5c, d). Therefore, either genetic or pharmacological inhibition of the NLRP3-IL-1β pathway hampered LPS-induced neurotoxicity. We further conducted a rescue study by adding small amounts of recombinant IL-1β to NLRP3^−/−^ neuron-glial cultures at 12 h after LPS. Lacking NLRP3 prevented LPS-elicited loss of dopaminergic neurons, whereas adding IL-1β at 40 pg/ml resulted in about 50% dopaminergic neuron loss at 7 days in LPS-treated NLRP3^−/−^ neuron-glial cultures (Fig. 5e, f). Although the amount of mature IL-1β released to the supernatant are merely ~ 40 pg/ml (about 2.2 × 10^−13^ M) (Fig. 4b), this little amount of IL-1β is sufficient for causing long-lasing neuroinflammation and neuronal loss. The essential role of IL-1β in chronic neuroinflammation-mediated neuronal damage was further confirmed in animal studies. The gradual loss of nigral dopaminergic neurons is one of the cardinal features of PD. Ten months after a single injection of LPS, a 30% loss of nigral dopaminergic neuron was found in WT; however, there was no significant difference between saline and LPS treatment in NLRP3^−/−^ or IL-1R1^−/−^ mice (Fig. 6a–c).

**Fig. 7:**
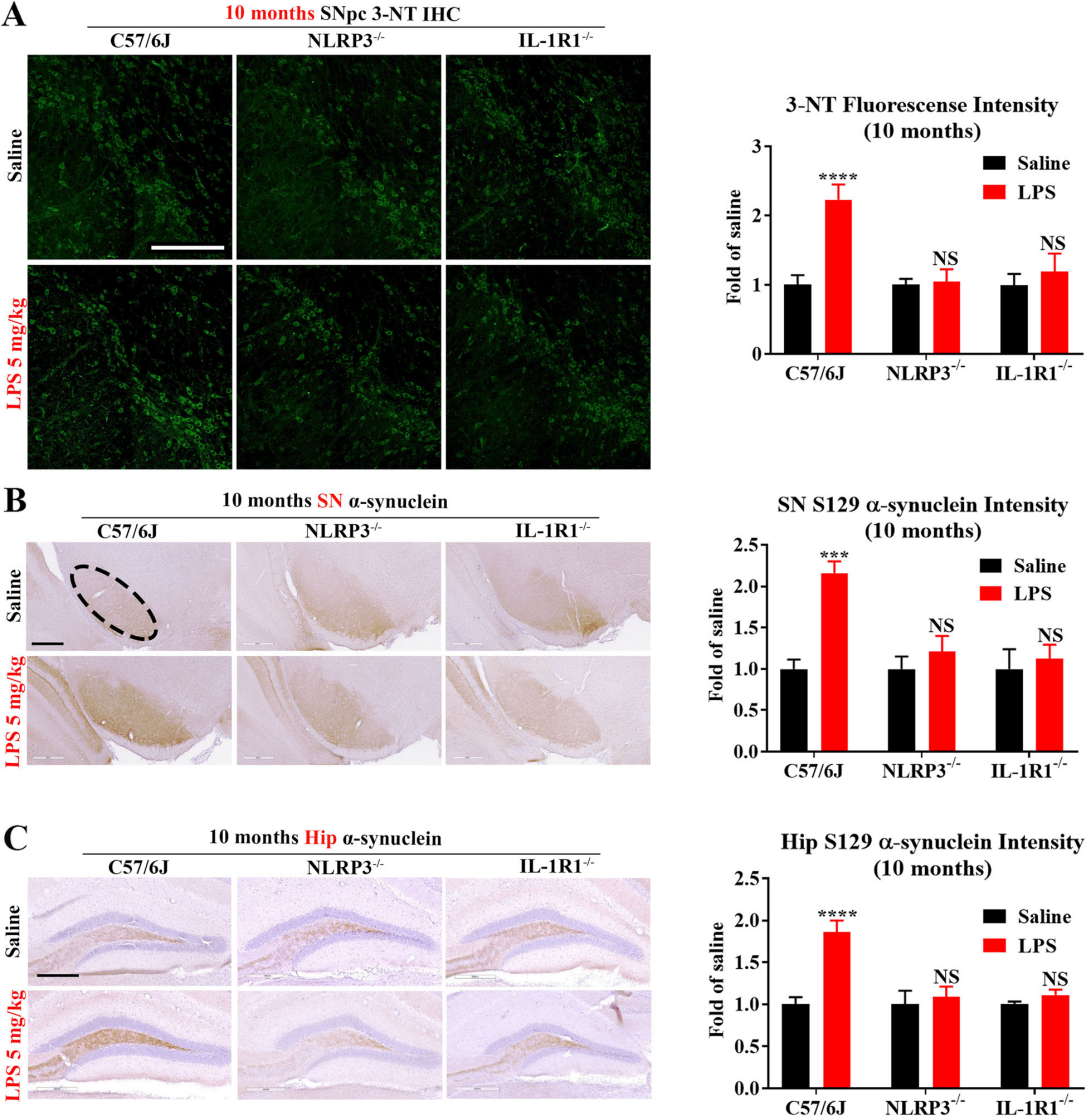
Peripheral LPS injection yields less protein nitrosylation and α-synuclein phosphorylation in NLRP3^−/−^ or IL-1R1^−/−^ mice. At 10 months after LPS (5 mg/kg, ip) or saline injection, representative images of 3NT immunofluorescence (green) in SN and the intensity of 3NT quantified with ImageJ were shown (**a**). *N* = 3/group. Bar = 300 μm. Representative images of Ser-129 phosphorylated α-synuclein staining in SN (**b**) and hippocampus (Hip) (**c**) and the intensity of Ser-129 phosphorylated α-synuclein staining (brownish staining in the mossy fibers) quantified with ImageJ were shown (S129 in the pictures means Ser-129 phosphorylated). *N* = 3/group. Scale bar = 400 μm (SN) or 300 μm (Hip). ***p < 0.001, ****p < 0.001, and NS compared with respective saline group. Two-way ANOVA followed by Bonferroni post hoc multiple comparison test

To provide evidence indicating that oxidative stress resulted from sustained chronic neuroinflammation underlies the mechanism of sepsis-associated long-term neurotoxicity, we measured changes of protein nitrosylation [68, 69] and α-synuclein phosphorylation at position Ser-129 [70], which are critical for the pathogenesis of PD [71–73]. Enhanced nitrosylation of proteins in the nigral region was observed in WT, but not in NLRP3^−/−^ or IL-1R1^−/−^, mice at 10 months after LPS injections (Fig. 7a and Additional file 4: Figure S4). Besides SN, aggregation of α-synuclein in PD patients can develop in different brain regions, such as the hippocampus [74]. Prominent increases in the immunoreactivity of Ser-129 phosphorylated α-synuclein in SN and hippocampus of WT mice at 10 months after LPS treatment was found. By contrast, LPS did not increase Ser-129 phosphorylated α-synuclein-ir in NLRP3^−/−^ or IL-1R1^−/−^ mice (Fig. 7b, c).

## Discussion

**Fig. 8:**
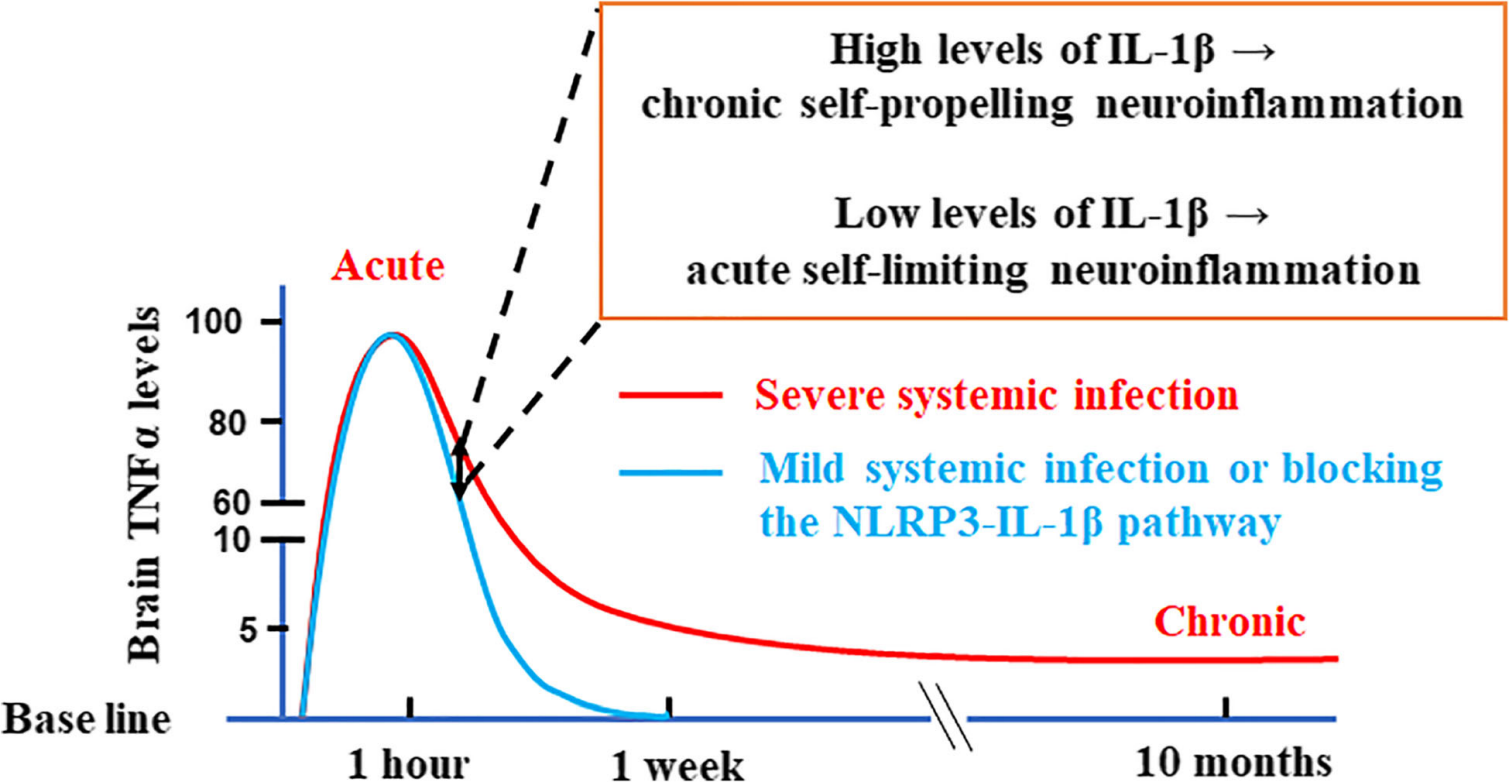
Schematic drawing depicting how brain IL-1β levels determine the fate of neuroinflammation induced by endotoxemia. Initial acute proinflammatory responses in the brain are similar between mild and severe endotoxemia; however, long-term outcomes are different. The curves of microglial activation are represented by TNFα concentrations in brain, because our previous study demonstrated that brain TNFα peaked at 1 h after LPS i.p. injection and then lasted at low levels in the endotoxemia-elicited neurodegeneration mouse model [17]. The NLRP3-IL-1β pathway gauges the severity of endotoxemia and generates dose-dependent increases of brain mature IL-1β around 7–11 h after LPS injection. Low levels of brain IL-1β permit the resolution of acute neuroinflammation as occurred in mild endotoxemia. In contrast, high levels of brain IL-1β during severe endotoxemia lead to chronic self-propelling neuroinflammation. This figure also suggests that in severe endotoxemia, reducing the production or hampering the function of brain IL-1β during the systemic acute inflammatory stage may provide a therapeutic window by preventing the transition of acute to chronic neuroinflammation and lowering the risk of resultant progressive neurodegeneration

Failure of immune resolution may lead to chronic brain inflammation and subsequent neurodegenerative diseases, such as Parkinson’s or Alzheimer’s diseases. Elucidation of the molecular mechanism mediating the transition from acute to chronic neuroinflammation is paramount to further understanding the pathogenesis of these neurodegenerative diseases. Here, we provide the first evidence indicating that NLRP3 inflammasome-generated IL-1β from microglia is the key cytokine in governing the transition of neuroinflammatory phases elicited by severe endotoxemia. NLRP3 gauges the severity of endotoxemia and determines the amount of mature IL-1β to produce from its precursor protein. Excessive release of IL-1β triggers the transition of acute to chronic neuroinflammation and eventually results in progressive neurodegeneration. Our study not only identified a novel role of IL-1β in the pathophysiology of endotoxemia-elicited neuroinflammation but also suggested a new strategy for developing potential therapies for neurodegenerative diseases. A schematic drawing depicting how brain IL-1β levels determine the fate of neuroinflammation induced by endotoxemia is shown in Fig. 8.

### Roles of IL-1β in neuroinflammation

Studies indicate that proinflammatory cytokines/chemokines, such as TNFα, IL-6, IL-1β, or MCP-1, etc., released of from activated microglia are important mediators for sepsis-associated brain inflammation [75]. This study unveiled a distinct role of IL-1β in the neuroinflammatory process. Unlike TNFα, which is necessary in triggering acute neuroinflammation [17], IL-1β is dispensable for the initiation of acute neuroinflammation but is essential for the launch of chronic neuroinflammation in mice with peripheral LPS administration.

Roles of IL-1β in mediating infection-induced acute sickness behaviors, the pathophysiological interactions between brain and immune systems, are well known [76–79]. However, information is limited regarding involvement of IL-1β in the long-term consequences in the brain after severe systemic infections. Previous reports indicate that IL-1β causes neurotoxicity per se or exacerbates pre-existing inflammatory response [80, 81]. However, the concentrations of IL-1β used in these studies were several orders of magnitude higher than the maximal amount that can be produced from brain. Thus, the pathophysiological function of IL-1β remains unclear. In this study, we provided strong evidence demonstrating a novel role of this cytokine in sepsis-induced long-term neuropathology. This conclusion was mainly based on the results from the following two studies.

#### High and low doses of peripheral LPS produce similar acute inflammation, but generate differential outcomes of sustained chronic inflammation and neurodegeneration

A dose-response study of LPS revealed that 1 and 5 mg/kg of LPS produced comparable proinflammatory cytokine induction and microglial activation in acute neuroinflammation (Fig. 1j). Interestingly, weeks or months after LPS injection, sustained chronic inflammation and neuronal loss were observed only in high doses of LPS-treated mice (Figs. 3 and 6). A unique pattern of brain IL-1β expression led us to believe that this cytokine plays a key role in sustaining high dose LPS-elicited chronic neuroinflammation. Despite no difference in both mRNA and precursor protein levels between high and low doses of LPS, we found that mature IL-1β was the only cytokine displaying a dose-dependent increase in the brain (Fig. 1d–i). As will be discussed below, production of larger amount of brain mature IL-1β is a critical link between higher degree of endotoxemia and chronic neuroinflammation.

#### Deficiency in NLRP3-IL-1β-IL-1R1 does not prevent the initiation of sepsis-associated acute neuroinflammation, but impedes long-term neuroinflammation and neurodegeneration

Production of most cytokines in immune cells is transcriptionally regulated; often translated precursor proteins are fully processed and mature cytokines are released extracellularly. But the production of mature IL-1β is an exception. Processing of precursor proteins to mature IL-1β is tightly regulated by inflammasomes, such as NLRP3 inflammasome; only a portion of the precursor is converted to mature IL-1β. This extra regulatory mechanism serves a critical function in gauging the severity of assaults.

We found that injection of LPS (5 mg/kg; i.p.) produced similar increases in the expression of several major brain proinflammatory cytokines, such as TNFα, IL-6 (1 h after LPS injection), and microglial activation (6 h after LPS injection) during the acute inflammatory phase in WT and mutant (NLRP3^−/−^, IL-1R1^−/−^) mice (Fig. 3a and Additional file 3: Figure S3). By contrast, LPS-induced chronic inflammatory response and progressive neuronal loss in certain brain regions were found in the WT mice, but not in mutant mice (Figs. 3b–d and 6).

Taken together, our findings strongly suggest that the NLRP3-IL-1β-IL-1R1 signaling does not participate in the initiation of acute neuroinflammation, rather the critical role of NLPR3-generated IL-1β is to gate the transition of acute to chronic neuroinflammation.

### A permissive role of IL-1β in switching acute self-limiting neuroinflammation to chronic self-propelling neuroinflammation

As mentioned above, the pathophysiological function of IL-1β during the process of infection-related inflammation in the brain remains unclear. Several observations from this study suggest a permissive role for IL-1β in switching acute self-limiting neuroinflammation to chronic self-propelling neuroinflammation. Although IL-1β is considered as a proinflammatory cytokine, our study suggests that it is unlikely that IL-1β directly participates in the formation of acute inflammation. First, blockade of NLRP3-IL-1β-IL-1R1 signaling does not prevent the initiation of LPS-induced acute inflammation (Additional file 3: Figure S3 and Fig. 3a). Second, the amount of IL-1β produced by LPS either in cell cultures or in mice brain is several orders of magnitude lower than that of most proinflammatory immune factors, such as TNFα, IL-6, MCP-1, etc. Our study showed that adding 80 pg/ml of recombinant IL-1β alone, which is two times higher than what is measured in vitro, to neuron-glial cultures failed to produce neuronal damage. Instead, adding 40 pg/ml (2.2 × 10^−13^ M) of IL-1β to LPS-treated neuron-glial cultures from NLRP3-deficient mice fully reinstated LPS-induced progressive dopaminergic neuron toxicity (Fig. 5). Third, time course studies showed that the release of IL-1β in LPS-simulated cell cultures was behind the other proinflammatory immune factors and the period of enhanced release of IL-1β was short-lived (Fig. 1). Thus, our study strongly suggests that depending on the severity of challenge, a corresponding amount of IL-1β was strategically released at a critical time point to orchestrate the transition of acute to chronic phase during the process of infection-related inflammation in the brain. Additionally, the permissive role for IL-1β in switching neuroinflammatory phases is supported by the repression of LPS-induced upregulation of late-expressed genes, such as Mac1, NOX2, and MHC-II, in the absence of NLRP3-IL-1β signal (Fig. 4).

Aggregated α-synuclein, one of PD hall markers, triggers microglial NLRP3 inflammasome and the downstream effector caspase-1 to release mature IL-1β [82]. In turn, activated caspase-1 causes truncation and aggregation of α-synuclein [83]. Moreover, inhibition of NLRP3 inflammasome was found to prevent α-synuclein pathology [84]. It seems that the interplay between aggregated α-synuclein and NLRP3 inflammasome is a component of the vicious cycle between damaged neuron and reactivated microglia, which is necessary for maintaining chronic self-propelling neuroinflammation. The finding that α-synuclein pathology was greatly diminished in LPS-injected NLRP3 or IL-1R1 KO mice in the present study (Fig. 7) adds further evidence indicating the interaction between α-synuclein and NLRP3 inflammasome.

### The NLRP3-IL-1β-IL-1R1 axis as a potential target for new drug therapy

Experimental or clinical studies show promising therapeutic results by targeting IL-1β signaling for a broad spectrum of disorders including cardiovascular disease and cancer treatment-associated life-threatening cytokine release syndrome [85–88]. From a clinical viewpoint, our study also provides valuable insights identifying the NLRP3-IL-1β-IL-1R1 axis as potential targets for developing new drug therapies for preventing severe infection-elicited chronic inflammation and neurodegeneration. In line with this possibility, MCC950, a small molecule of NLRP3 inflammasome inhibitor, capable of gaining access to the brain, was reported to exert neuronal protective effects in several animal PD models [84]. It is notable that, besides neurotoxicity, IL-1β also plays neuroprotective function under certain experimental conditions. For example, IL-1β was found to participate in both the classical and alternative activation of microglia in a in vivo spinal cord injury mouse model [89]. In vitro, expression of the alternative activation markers arginase-1 and Ym1 was increased in primary microglial cultures after IL-4 stimulation and further increased after co-treatment with IL-4 and IL-1β. Therefore, cautions must be contextually exercised when considering the application of therapeutic strategies to target IL-1β.

Moreover, astroglia can express NLRP3 inflammasome and IL-1β under certain conditions, such as in SOD1 mouse model of amyotrophic lateral sclerosis (ALS) and human sporadic ALS patients, and amyloid β_1–42_-stimulated murine astrocytes [90, 91]. We could not rule out the role of astroglia in LPS-induced sepsis-associated neurodegeneration. MCC950 also displayed the potency to repress astroglial NLRP3 inflammasome [91]. These findings highlight the promising application of NLRP3-IL-1β signaling inhibition in various neurodegenerative disorders.

## Conclusions

This study uncovers a novel role of the NLRP3-IL-1β signaling pathway in gauging the severity of sepsis-associated inflammation and determining whether acute neuroinflammation will resolve or transition to low grade chronic neuroinflammation. These findings also provide novel targets for developing therapy for severe systemic infection-related neurodegeneration.

## Supplementary information

**Additional file 1: Figure S1.** Lack of dose-response of LPS-elicited production of IL-1β precursor in the brain. Representative images of western blot analysis of IL-1β precursor in C57BL/6 J mice brain tissue at indicated time after LPS 1 or 5 mg/kg ip injection. Quantification of the western blot was presented in Fig. 1e.

**Additional file 2: Figure S2.** ER stress mediates LPS-induced processing of microglial precursor to mature IL-1β. (a) NLRP3 mRNA was measured by qPCR at indicated time points in mix-glial cultures after LPS 10^3^ ng/ml treatment. Results were from 3 independent experiments performed in duplicate. (b) and (c) YVAD, TUDC and 4μ8C were administrated at indicated concentrations in mix-glial cultures at 6 h after LPS 10^3^ ng/ml treatment. Culture supernatant levels of IL-1β were measured at 24 h. Results were from 3 independent experiments. ****p < 0.0001 compared to vehicle group and #p < 0.0001 compared to 10^3^ng/ml group. One-way ANOVA followed by Bonferroni post hoc multiple comparison test.

**Additional file 3: Figure S3.** Deficiency in NLRP3 or IL-1R1 does not prevent brain initial acute inflammatory response. At 1 h after injection of LPS (5 mg/kg, ip) or saline vehicle in C57BL/6 J mice, brain mRNA levels of TNFα (a), IL-6 (b), MCP-1 (c), and IL-1β (d) were measured by qPCR (*n* = 4/group). ***p < 0.001 compared with respective saline vehicle group. Two-way ANOVA followed by Bonferroni post hoc multiple comparison test.

**Additional file 4: Figure S4.** Peripheral LPS injection enhances protein nitrosylation in WT but not mutant mice. Representative images of TH (red) and 3-NT/TH double (yellow) immunofluorescence double staining in SN of WT, NLRP3^−/−^, and IL-1R1^−/−^ mice at 10 months after LPS 5 mg/kg or saline i.p injection (*n* = 3/group). Bar = 300 μm. The 3-NT staining pictures and the quantification of 3-NT intensity were shown in Fig. 7.

## Data Availability

All data generated or analyzed during this study are included in this article.

## Abbreviations

3-NT: 3-Nitrotyrosine
ER: Endoplasmic reticulum
Iba-1: Ionized calcium binding adaptor molecule 1
IL-1R1: IL-1 receptor 1
IL-1Ra: Interleukin-1 receptor antagonist
IL-1α/1β/6: Interleukin-1α/1β/6
ip: Intraperitoneal
IRE1α: Inositol-requiring enzyme 1α
LPS: Lipopolysaccharide
MCP-1: Monocyte chemoattractant protein-1
MHC-II: Major histocompatibility complex II
NLRP3: Nod-like receptor protein 3
NO: Nitric oxide
PD: Parkinson’s disease
ROS: Reactive oxygen species
TNFα: Tumor necrosis factor alpha
