# Supplementary figures and images for: A novel role of NLRP3-generated IL-1β in the acute-chronic transition of peripheral lipopolysaccharide-elicited neuroinflammation: implications for sepsis-associated neurodegeneration
Source: J Neuroinflammation. 2020 Feb 18;17:64. doi: 10.1186/s12974-020-1728-5 (PMC7029475; doi:10.1186/s12974-020-1728-5)

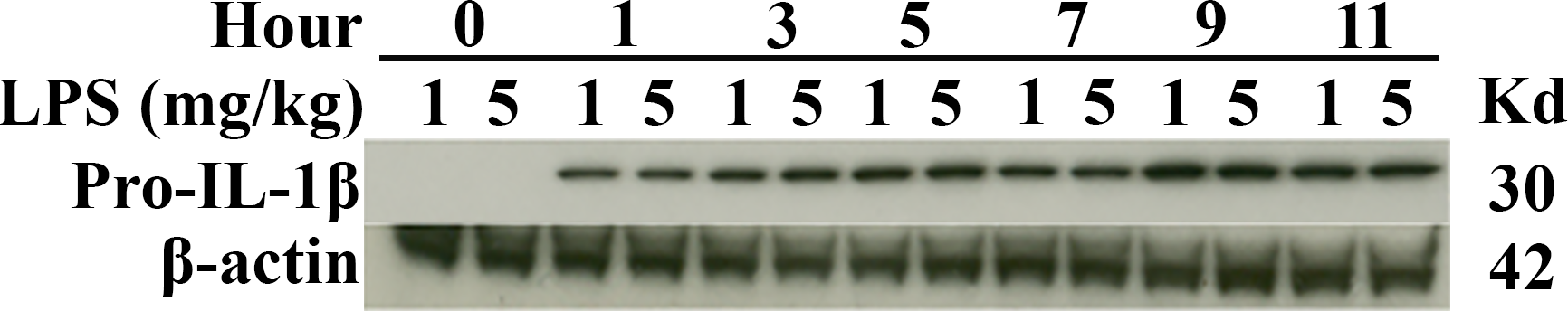

Supplement: Supplementary file 1 — Additional file 1: Figure S1. Lack of dose-response of LPS-elicited production of IL-1β precursor in the brain. Representative images of western blot analysis of IL-1β precursor in C57BL/6 J mice brain tissue at indicated time after LPS 1 or 5 mg/kg ip injection. Quantification of the western blot was presented in Fig. 1e. [file 12974_2020_1728_MOESM1_ESM.tif]

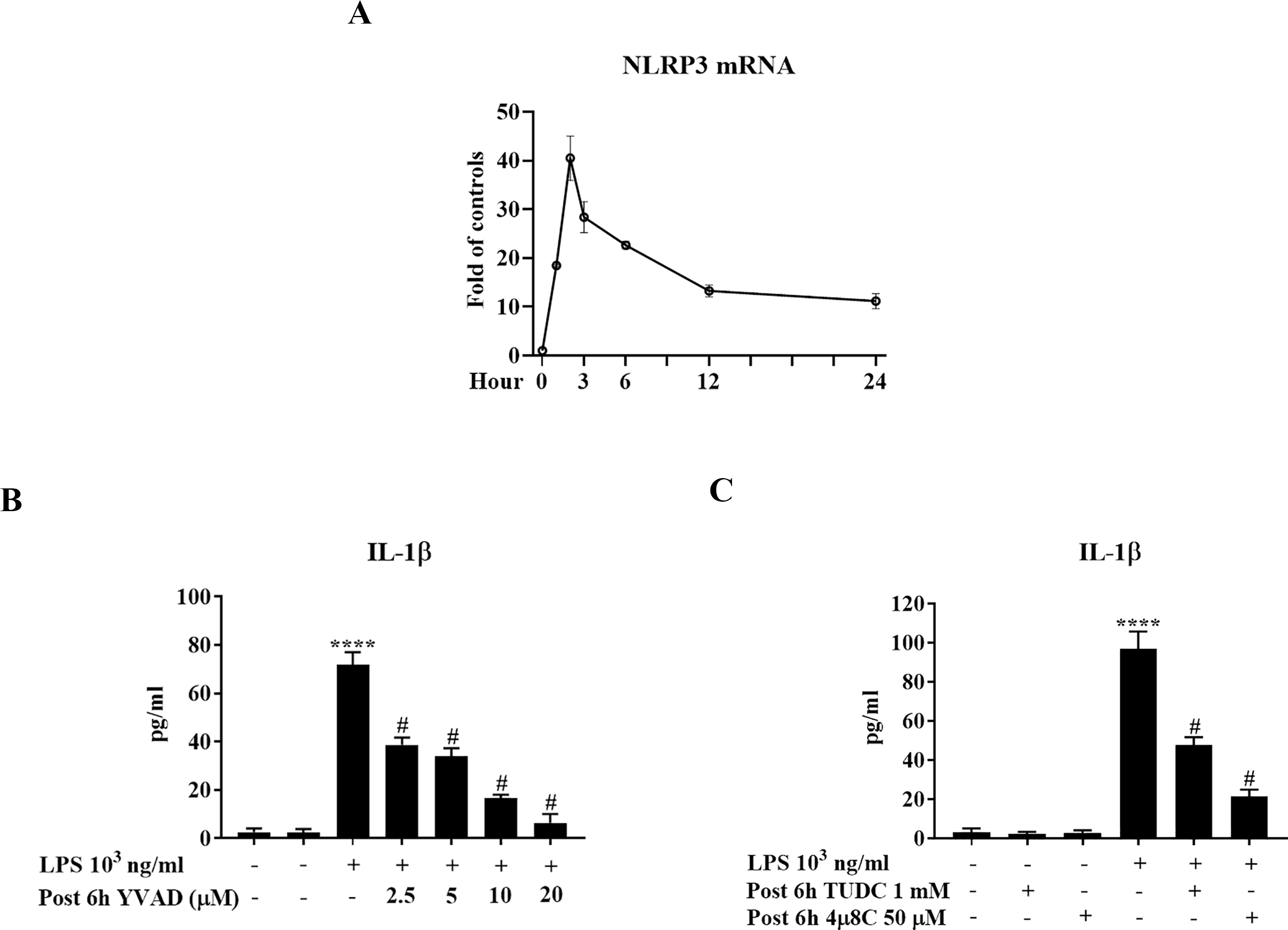

Supplement: Supplementary file 2 — Additional file 2: Figure S2. ER stress mediates LPS-induced processing of microglial precursor to mature IL-1β. (a) NLRP3 mRNA was measured by qPCR at indicated time points in mix-glial cultures after LPS 103 ng/ml treatment. Results were from 3 independent experiments performed in duplicate. (b) and (c) YVAD, TUDC and 4μ8C were administrated at indicated concentrations in mix-glial cultures at 6 h after LPS 103 ng/ml treatment. Culture supernatant levels of IL-1β were measured at 24 h. Results were from 3 independent experiments. ****p < 0.0001 compared to vehicle group and #p < 0.0001 compared to 103ng/ml group. One-way ANOVA followed by Bonferroni post hoc multiple comparison test. [file 12974_2020_1728_MOESM2_ESM.tif]

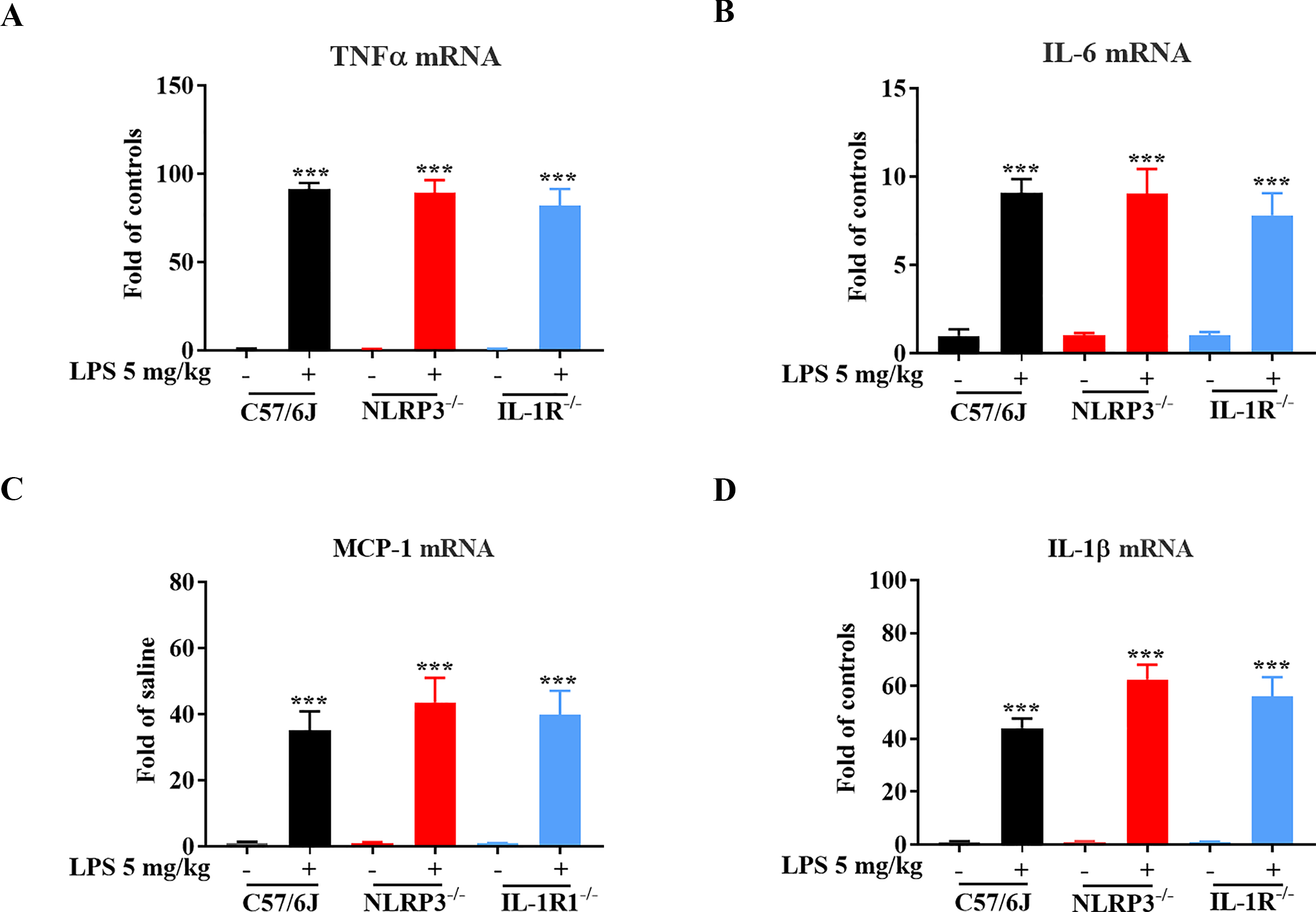

Supplement: Supplementary file 3 — Additional file 3: Figure S3. Deficiency in NLRP3 or IL-1R1 does not prevent brain initial acute inflammatory response. At 1 h after injection of LPS (5 mg/kg, ip) or saline vehicle in C57BL/6 J mice, brain mRNA levels of TNFα (a), IL-6 (b), MCP-1 (c), and IL-1β (d) were measured by qPCR (n = 4/group). ***p < 0.001 compared with respective saline vehicle group. Two-way ANOVA followed by Bonferroni post hoc multiple comparison test. [file 12974_2020_1728_MOESM3_ESM.tif]

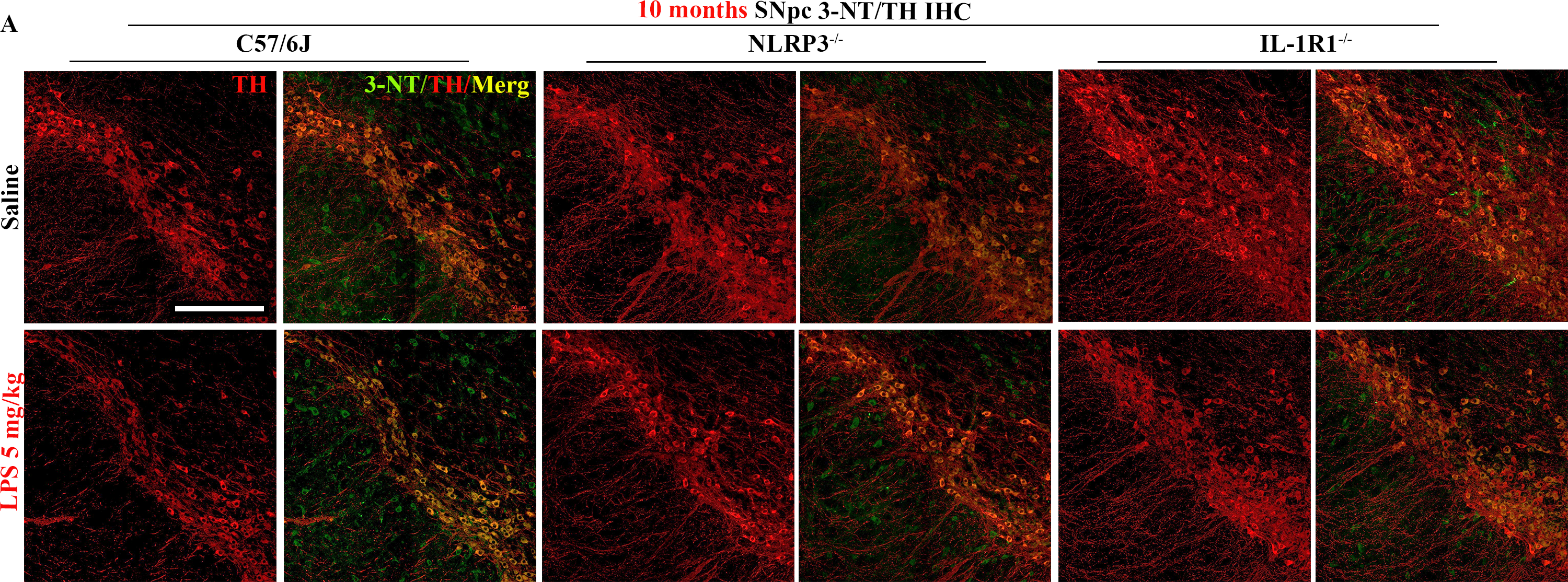

Supplement: Supplementary file 4 — Additional file 4: Figure S4. Peripheral LPS injection enhances protein nitrosylation in WT but not mutant mice. Representative images of TH (red) and 3-NT/TH double (yellow) immunofluorescence double staining in SN of WT, NLRP3−/−, and IL-1R1−/− mice at 10 months after LPS 5 mg/kg or saline i.p injection (n = 3/group). Bar = 300 μm. The 3-NT staining pictures and the quantification of 3-NT intensity were shown in Fig. 7. [file 12974_2020_1728_MOESM4_ESM.tif]
